# Metabolomic Biomarkers to Predict and Diagnose Cystic Fibrosis Pulmonary Exacerbations: A Systematic Review

**DOI:** 10.3389/fped.2022.896439

**Published:** 2022-05-31

**Authors:** Anna-Lisa V. Nguyen, Dominic Haas, Mégane Bouchard, Bradley S. Quon

**Affiliations:** ^1^Centre for Heart Lung Innovation, University of British Columbia, Vancouver, BC, Canada; ^2^Faculty of Health Sciences, McMaster University, Hamilton, ON, Canada; ^3^Department of Epidemiology, Biostatistics, and Occupational Health, McGill University, Montréal, QC, Canada; ^4^Faculty of Medicine, University of British Columbia, Vancouver, BC, Canada

**Keywords:** metabolomics, biomarkers, systematic review, respirology, cystic fibrosis

## Abstract

**Introduction:**

Metabolomics is an emerging area of research and has the potential to identify clinical biomarkers for predicting or diagnosing cystic fibrosis (CF) pulmonary exacerbations (PEx).

**Objective:**

To identify clinically promising metabolites across different sample sources that can be used to predict or diagnose PEx in CF.

**Evidence Review:**

Searches for original literature were completed through EMBASE, MEDLINE, and all databases on the Web of Science with no restrictions on language or publication date. Gray literature was collected through Google Scholar. Additional studies were obtained by contacting authors and searching reference lists of candidate papers. The patient population included individuals with CF. Studies involving patients who underwent lung transplantation were excluded. The outcome was the prediction or diagnosis of pulmonary exacerbations from metabolites directly measured from biological samples. Search results were downloaded and imported into Covidence and duplicates were removed automatically. Any remaining duplicates were manually tagged and excluded. Two independent reviewers screened each abstract for eligibility and repeated this process for full texts. Risk of bias was conducted using QUADAS-2 by two independent reviewers. A third author resolved any remaining conflicts.

**Results:**

A combined 3974 relevant abstracts were identified and 115 full texts were assessed for eligibility. The final 25 studies underwent data extraction for study design, patient demographics, studied metabolites, concentration values, and diagnostic accuracy values. Included studies differed considerably in methodologies, sample specimen types (exhaled breath condensate [EBC], sputum, saliva, plasma, urine), and disease states. We identified 19 unique metabolites that were measured by two or more studies of which 2 have the potential to predict PEx (EBC 4-hydroxycyclohexylcarboxylic acid [4-HCHC] and lactic acid) and 6 to diagnose PEx (EBC 4-HCHC and lactic acid, sputum lactic acid and nitrate, and plasma arginine and methionine).

**Conclusion and Relevance:**

This systematic review has identified promising metabolites for further study in CF. Certain metabolites may provide clinical potential in predicting or diagnosing PEx, but further validation studies are required. With better tools to aid in the earlier identification of PEx, clinicians can implement preventative measures to mitigate airway damage.

**Systematic Review Registration:** https://www.crd.york.ac.uk/prospero/

## Introduction

Cystic fibrosis (CF) is one of the most common inherited life-shortening conditions that causes chronic progressive lung disease. The disease course is characterized by episodes of acute or subacute clinical worsening referred to as pulmonary exacerbations (PEx) due to increased airway infection and inflammation. PEx symptoms typically include increased productive cough and systemic symptoms such as fatigue, loss of appetite, and weight loss (1). The clinical presentation with PEx can be subtle, especially early in its course, resulting in missed opportunities to intervene with antibiotics to preserve lung function. By using biomarkers to predict imminent exacerbation risk or facilitate earlier diagnosis, patient outcomes can be improved.

Within the “-omics” field, most of the focus has been on examining sputum and blood proteomic and transcriptomic inflammatory markers in CF patients to diagnose PEx (2, 3). However, metabolomic studies are also emerging and involve a range of biospecimens. While most studies have focused on differences in metabolites between CF and non-CF subjects, several studies have also focused on PEx. The present systematic review has synthesized data from the available literature to determine if there are promising metabolites across the various biospecimens to be evaluated further as clinical biomarkers in the prediction and diagnosis of PEx.

## Materials and Methods

### Study Eligibility

To define the review question, the PICO framework for diagnostic tests was implemented (4). Our population of interest was patients who had a confirmed diagnosis of CF. The investigated test of interest was the analysis of metabolites as a predictive or diagnostic biomarker. A comparator test was not applicable for this review. The outcome was the prediction or diagnosis of a PEx based on various research criteria, such as Fuchs, Rosenfeld, or physician decision to begin antibiotic treatment (5, 6).

Studies that collected biospecimens for the purpose of analyzing metabolites were included in this review. We broadly included studies that used metabolites to predict or diagnose PEx in CF patients. Studies that only examined metabolite changes throughout PEx treatment were excluded. Furthermore, studies were excluded if they solely focused on laboratory samples (e.g., *in vitro* studies). Studies were also excluded if they used genomic, proteomic, or microbiome approaches without addressing metabolites. A detailed summary of study characteristics is presented in Table 1.

**Table 1:** Study design details for the included studies.

**Author**	**Country**	**Single or multi-center**	**Paired or independent groups**	**HC vs. SCF**	**# Total participants (HC, SCF, PEx)**	**Adult, pediatric, or both**	**Age Mean (SD) Median [IQR]**	**PEx FEV_**1**_ (%) Mean (SD) Median [IQR]**	**PEx diagnostic criteria**
Alvarez et al. (7)	United States	Single	Independent	HC	52 (28, 0, 24)	Adult	28.5 (7.5)	45.0 (31.0)	P
Barr et al. (8)	UK	Multi-center	Paired	SCF	89 (0, 29, 60)	Adult	29.3 (10.4)	47.2 (16.9)	R
Cantin et al. (9)	Canada	Single	Paired and separate (subgroups of larger cohort were paired)	Both	75 (47, 16, 12)	Adult	15.1 (8.1)	—	R
Felton et al. (10)	United States	Single	Paired	SCF	27 (0, 0, 27)	Pediatric	10.0 (—)	81.7 (19.4)	R
Ghorbani et al. (11)	Canada	Single	Independent	SCF	20 (0, 11, 9)	Pediatric	13.4 (2.7)	52.9 (11.9)	—
Grasemann et al. (12)	Germany	Single	Independent	Both	92 (53, 18, 21)	Both	19.6 (8.6)	35.9 (16.0)	R
Grasemann et al. (13)	Canada	Single	Independent	HC	20 (10, 0, 10)	Both	23.2 (4.2)	32.4 (10.6)	P
Grasemann et al. (14)	Canada	Single	Independent	Both	45 (11, 16, 18)	Pediatric	13.7 (—)	52.5 (—)	P
Grasemann et al. (15)	Canada	Single	Independent	Both	40 (10, 10, 20)	Both	14.8 (2.9)	53.1 (—)	—
Hanusch et al. (16)	Germany	Single	Independent	Both	148 (78, 24, 46)	Pediatric	11.7 [8–14]	81.7 (18.3)	S
Ho et al. (17)	UK	Single	Independent	Both	46 (0, 36, 10)	Adult	26.8 (0.3)	—	S
Lagrange-Puget et al. (18)	France	Multi-center	Paired	Both	312 (53, 312, 312)^a^	Both	16.0 (—)	—	P
Linnane et al. (19)	Ireland	Single	Independent	Both	47 (9, 13, 25)	Adult	24.7 (4.6)	34.0 (10.0)	—
Lucca et al. (20)	Italy	Single	Independent	SCF	50 (16, 21, 13)	Pediatric	14.2 (3.1)	80.8 (10.2)	P
McGrath et al. (21)	UK	Single	Independent	Both	24 (12, 0, 12)	Adult	25.0 (—)	1.6 (0.3)^*^^†^	S
Montuschi et al. (22)	Italy	Single	Independent	Both	84 (31, 29, 24)	Both	14.7 (0.8)	75.7 (3.4)^*^	S
Quinn et al. (23)	United States	Single	Paired	SCF	6 (0, 6, 6)^a^	Adult	—	—	R
Raghuvanshi et al. (24)	United States	Single	Paired	SCF	6 (0, 6, 6)^a^	Adult	32.7 (7.8)	—	S
Topcu et al. (25)	Turkey	Single	Independent	Both	45 (17, 9, 19)	Pediatric	11.3 (3.0)	61.0 (26.9)	S
Twomey et al. (26)	UK	Single	Independent	SCF	80 (5, 0, 75)	Adult	28.3 (—)	—	R
vanHorck et al. (27)	Netherlands	Multi-center	Paired	SCF	49 (0, 11, 38)	Pediatric	10.3 (3.6)	—	R
Vazquez et al. (28)	United States	Single	Independent	SCF	28 (—)	—	—	—	—
Wojewodka et al. (29)	Canada	Single	Paired	Both	52 (0, 15, 37)	Both	32.8 (1.8)	56.8 (4.6)^*^	S
Zang et al. (30)	United States	Single	Paired	SCF	26 (0, 17, 9)	Both	27.0 (8.0)	—	S, P
Zang et al. (31)	United States	Single	Paired	SCF	138 (0, 97, 41)	Both	26.8 (—)	—	R

a *Paired samples were used in both states*.

*HC, Healthy controls; SCF, Stable CF; P, Physician-defined criteria; R, Researched diagnostic criteria (e.g., Fuchs, Rosenfeld, etc.); S, Study-specific criteria; SD, Standard deviation; IQR, Interquartile range; vs, versus*.

* *Reported in standard error of the mean (SEM)*.

† *Reported in liters (L)*.

### Search Strategy

The search strategy and protocol were registered through PROSPERO [CRD42021269309]. Our study followed the Preferred Reporting Items for Systematic reviews and Meta-Analyses (PRISMA) guidelines for the search strategy, text screening, and data extraction (32). A health sciences librarian advised on the search strategy throughout the piloting process. The primary author (AVN) conducted the last search on August 26, 2021. An example of the search terms used and a completed search can be found in Supplementary Table 1 (Appendix). Alerts for newly published literature were sent to the primary author. Searches for original literature were completed through MEDLINE *via* Ovid, EMBASE *via* Ovid, and all databases on Web of Science with no restrictions on language, publication date, or publication status. The search strategy was adapted for each database. Gray literature was collected through Google Scholar. The authors decided to arbitrarily limit citations on Google Scholar to the first 200 results based on recommendations by Haddaway et al. (33). Case reports and case series were excluded from the review.

### Data Collection Process

A data collection form was created and modified based on recommendations from the PRISMA guidelines and included study details such as study design, patient demographics, metabolite processing methodology, statistical analysis methodology, sample source, identifiable metabolite nomenclature, metabolite concentration data, diagnostic accuracy data, association to inflammation, and risk of bias using Quality Assessment of Diagnostic Accuracy Studies (QUADAS-2) (34). The data extraction form was piloted on five studies and adjusted accordingly. Two independent reviewers (AVN and DH) extracted data from the eligible full-texts and proceeded to reach consensus through discussion and input from a third reviewer (BSQ).

### Synthesis of Results

Due to the heterogeneity of the included studies, a meta-analysis was not feasible. Details of the included studies are demonstrated in Table 1, including study designs and patient characteristics. A tabulated summary of studied metabolites that were classified based on their chemical taxonomy is presented in Table 2. The biospecimen types and analytical platforms used to measure the metabolites are detailed in Table 3. Furthermore, we included information on whether these unique metabolites were used to predict or diagnose PEx, the sample source, the direction of change in concentrations between disease states, and any statistical significance in comparisons between disease states. Metabolites that appeared in two or more studies are summarized in Table 4. Metabolite ratios and broad pathways implicated in PEx that were identified within studies were not included in our summary. A summary of the risk of bias using QUADAS-2 can be accessed in Supplementary Table 2 (Appendix).

**Table 2:** Metabolites evaluated in the included studies.

**Class**	**Biomarker(s)**
Benzoic acids and substituted derivatives	Hippurate (7)
Carboxylic acids and derivatives	Acetic acid/acetate (22, 31), ADMA (asymmetric dimethylarginine) (14, 16), alanine (13), arginine (7, 13, 16), asparagine (13), citrulline (13, 16), cysteine (13), desmosine, glutamic acid/glutamate (7, 13), glutamine (7), glutathione (18), glycine (13), histidine (7, 13), isoleucine (13), kynurenine (28), leucine (13), lysine (7, 13), methionine (7, 13), ornithine (13), phenylalanine (7, 13), proline (7, 13), prolylhydroxyproline (31), pyroglutamic acid/oxoproline (7, 30, 31), SDMA (symmetric dimethylarginine) (14), serine (13), sulphydryls (21), threonine (7, 13, 28), tryptophan (7, 13), tyrosine (7, 13), valine (13)
Diazines	Dihydrothymine (31)
Dihydrofurans	Ascorbic acid/ascorbate (21)
Fatty acyls	3-methylglutaconic acid (31), 2-methylglutaconic acid (31), 2-hexenedioic acid (31), 3-hexenedioic acid (31), nonanedioic acid/azelaic acid (31), sebacic acid (31), palmitate (26), rhamnolipids (24)
Glycerophosphocholines	Diacylglycerophosphocholine lipid PC (18:0/3:1) (23)
Hydroxy acids and derivatives	Lactic acid/lactate (26, 30, 31)
Imidazopyrimidines	Hypoxanthine (7), uric acid (7)
Keto acids and derivatives	Levulinic acid (31), pyruvate (26)
Lactones	γ-butyrolactone/oxolan-3-one (31)
Non-metal oxoanionic compounds	Nitrate (12, 16), nitrite (12, 16)
Organonitrogen compounds	Carnitine (7), putrescine (15, 26), spermidine (15), spermine (15)
Organooxygen compounds	4-hydroxycyclohexylcarboxylic acid (30, 31), 4-hydroxycyclohexylacetic acid (31), acetone (22), docosahexaenoic acid (DHA) (29), glucose (7), lipid peroxides (18), malondialdehyde (MDA) (18, 21), sialic acid (9)
Other non-metal organides	Nitric oxide (NO) (17, 19)
Prenol lipids	α-carotene (18, 21), β-carotene (18, 21), α-tocopherol (21), γ-tocopherol (21), lycopene (18, 21), retinal (21), retinol/vitamin A (18), vitamin E (18), ubiquinol 10 (21), zeaxanthine (18)
Quinolines and derivatives	4-hydroxy-2-heptyl quinolone (24), 4-hydroxy-2-nonylquinolone (NHQ) (24)

**Table 3:** Metabolite sample sources and analytical methodologies.

**Author**	**Sample source(s)**	**Analytical platform(s) used**	**Fasted status**
Alvarez et al. (7)	Plasma	LC-MS	No
Barr et al. (8)	Sputum, blood, urine	LC-MS/MS	—
Cantin et al. (9)	Plasma	Colorimetric assay	—
Felton et al. (10)	Sputum	DNA quantification	—
Ghorbani et al. (11)	Sputum	GC	—
Grasemann et al. (12)	Sputum, saliva	Colorimetric assay	—
Grasemann et al. (13)	Plasma	ELISA, ion exchange chromatography	Yes
Grasemann et al. (14)	Sputum	LC-MS	—
Grasemann et al. (15)	Sputum	HPLC, LC-MS/MS	—
Hanusch et al. (16)	Plasma, urine, sputum	GC-MS, GC-MS/MS, HPLC	No
Ho et al. (17)	EBC	Chemiluminescence analysis	—
Lagrange-Puget et al. (18)	Plasma	HPLC, HPLC-UV	—
Linnane et al. (19)	Sputum	Chemiluminescence analysis	—
Lucca et al. (20)	EBC	UPLC-ESI-MS/MS	—
McGrath et al. (21)	Plasma	HPLC	—
Montuschi et al. (22)	EBC	H-NMR, TOCSY	Yes
Quinn et al. (23)	Sputum	LC-MS/MS	—
Raghuvanshi et al. (24)	Sputum	UPLC	—
Topcu et al. (25)	Plasma	LC-MS	—
Twomey et al. (26)	Sputum	LC-MS/MS, HPLC	—
van Horck et al. (27)	EBC	GC-MS	—
Vazquez et al. (28)	Plasma	HPLC	—
Wojewodka et al. (29)	Plasma	GC-MS, ELISA	—
Zang et al. (30)	EBC	LC-MS, UPLC-MS	—
Zang et al. (31)	EBC	CCS, MS/MS, UPLC-MS	—

*EBC, Exhaled breath condensate; CCS, Collision cross-section; ELISA, Enzyme-linked immunosorbent assay; GC, Gas chromatography; H-NMR, Proton nuclear magnetic resonance; LC, Liquid chromatography; MS, Mass spectroscopy; HPLC, High performance liquid chromatography; UPLC, Ultra performance liquid chromatography; TOCSY, Total correlation spectroscopy spectra*.

**Table 4:** Metabolites analyzed in two or more studies from various biospecimens to predict or diagnose PEx.

				**PEx metabolite levels relative to healthy controls or stable CF**	
**Metabolites**	**Sample source**	**Study**	**Predictive or Diagnostic**	**PEx vs. Healthy controls**	**PEx vs. Stable CF**
Arginine	EBC	Zang et al. (31)	Predictive		Higher (paired)^a^
	Sputum	Hanusch et al. (16)	Diagnostic		Higher
	Plasma	Hanusch et al. (16)	Diagnostic		Lower
	Plasma	Alvarez et al. (7)	Diagnostic	Lower	
	Plasma	Grasemann et al. (13)	Diagnostic	Lower^*^	
ADMA (extracellular)	Sputum	Grasemann et al. (14)	Diagnostic	Higher	Higher
ADMA (intracellular)	Sputum	Grasemann et al. (14)	Diagnostic	Higher	Higher
ADMA	Sputum	Hanusch et al. (16)	Diagnostic		No change
	Plasma	Hanusch et al. (16)	Diagnostic		No change
	Urine	Hanusch et al. (16)	Diagnostic		No change
Carnitine	EBC	Zang et al. (31)	Diagnostic		Higher (paired)
	Plasma	Alvarez et al. (7)	Diagnostic	Higher^*^	
Citrulline	Plasma	Grasemann et al. (13)	Diagnostic	Higher	
	Plasma	Hanusch et al. (16)	Diagnostic		Lower
Glutamate/glutamic acid	EBC	Zang et al. (31)	Predictive		Higher (paired)^a^
	Plasma	Grasemann et al. (13)	Diagnostic	Higher^**^	
	Plasma	Alvarez et al. (7)	Diagnostic	No change	
Histidine	Plasma	Alvarez et al. (7)	Diagnostic	Lower^**^	
	Plasma	Grasemann et al. (13)	Diagnostic	Lower	
4-hydroxycyclohexylcarboxylic acid (4-HCHC)	EBC	Zang et al. (31)	Predictive		Higher (paired)^a^
	EBC	Zang et al. (30)	Diagnostic		Lower (paired)
					
	EBC	Zang et al. (31)	Diagnostic		Higher (paired)^**^
Lactic acid/lactate	EBC	Zang et al. (30)	Predictive		Higher (paired)^*^^a^
	EBC	Zang et al. (31)	Predictive		Higher (paired)^a^
	EBC	Zang et al. (31)	Diagnostic		Higher (paired)^**^
	Sputum	Twomey et al. (26)	Diagnostic		Higher^†^
Lysine	EBC	Zang et al. (31)	Predictive		Higher (paired)^a^
	Plasma	Alvarez et al. (7)	Diagnostic	Lower^*^	
	Plasma	Grasemann et al. (13)	Diagnostic	No change	
Malondialdehyde	Plasma	Lagrange-Puget et al. (18)	Diagnostic		Lower (paired)^†^
	Plasma	McGrath et al. (21)	Diagnostic	Higher	
Methionine	Plasma	Alvarez et al. (7)	Diagnostic		Lower^*^
	Plasma	Grasemann et al. (13)	Diagnostic		Lower
Nitric oxide (NO)	EBC	Linnane et al. (19)	Diagnostic	Lower^*^	No change
	EBC	Ho et al. (17)	Diagnostic	No change	7/10 same or lower than stable CF; 3/10 higher
Nitrate	Sputum	Hanusch et al. (16)	Diagnostic		Lower
	Sputum	Grasemann et al. (12)	Diagnostic	Higher	Lower
	Saliva	Grasemann et al. (12)	Diagnostic	Higher	Higher
	Plasma	Hanusch et al. (16)	Diagnostic		No change
	Urine	Hanusch et al. (16)	Diagnostic		No change
Nitrite	Sputum	Grasemann et al. (12)	Diagnostic		Lower
	Sputum	Hanusch et al. (16)	Diagnostic		Higher
	Saliva	Grasemann et al. (12)	Diagnostic	Higher	Lower
**Metabolites**	**Sample source**	**Study**	**Predictive or Diagnostic**	**PEx vs. Healthy controls**	**PEx vs. Stable CF**
	Plasma	Hanusch et al. (16)	Diagnostic		No change
	Urine	Hanusch et al. (16)	Diagnostic		No change
Phenylalanine	Plasma	Alvarez et al. (7)	Diagnostic	Lower	
	Plasma	Grasemann et al. (13)	Diagnostic	Lower	
Proline	EBC	Zang et al. (31)	Predictive		Lower (paired)^a^
	Plasma	Alvarez et al. (7)	Diagnostic	No change	
	Plasma	Grasemann et al. (13)	Diagnostic	No change	
					
Putrescine	Sputum	Grasemann et al. (15)	Diagnostic	Higher^*^	Higher
	Sputum	Twomey et al. (26)	Diagnostic	Higher	Higher^†^
Pyroglutamic acid, oxoproline	EBC	Zang et al. (30)	Predictive		Higher (paired)^*^^a^
	EBC	Zang et al. (31)	Diagnostic		Higher (paired)
	Plasma	Alvarez et al. (7)	Diagnostic	Lower^*^	
Tryptophan	EBC	Zang et al. (31)	Diagnostic		Lower
	Plasma	Grasemann et al. (13)	Diagnostic	Lower^**^	
	Plasma	Alvarez et al. (7)	Diagnostic	Lower^*^	

a *Examined using pre-PEx samples*.

* *p-value ≦ 0.05*.

** *p-value ≦ 0.01*.

† *p-value ≦ 0.001*.

*vs, versus; ADMA, asymmetric dimethylarginine; EBC, exhaled breath condensate*.

## Results

### Study Selection

**Figure 1:**
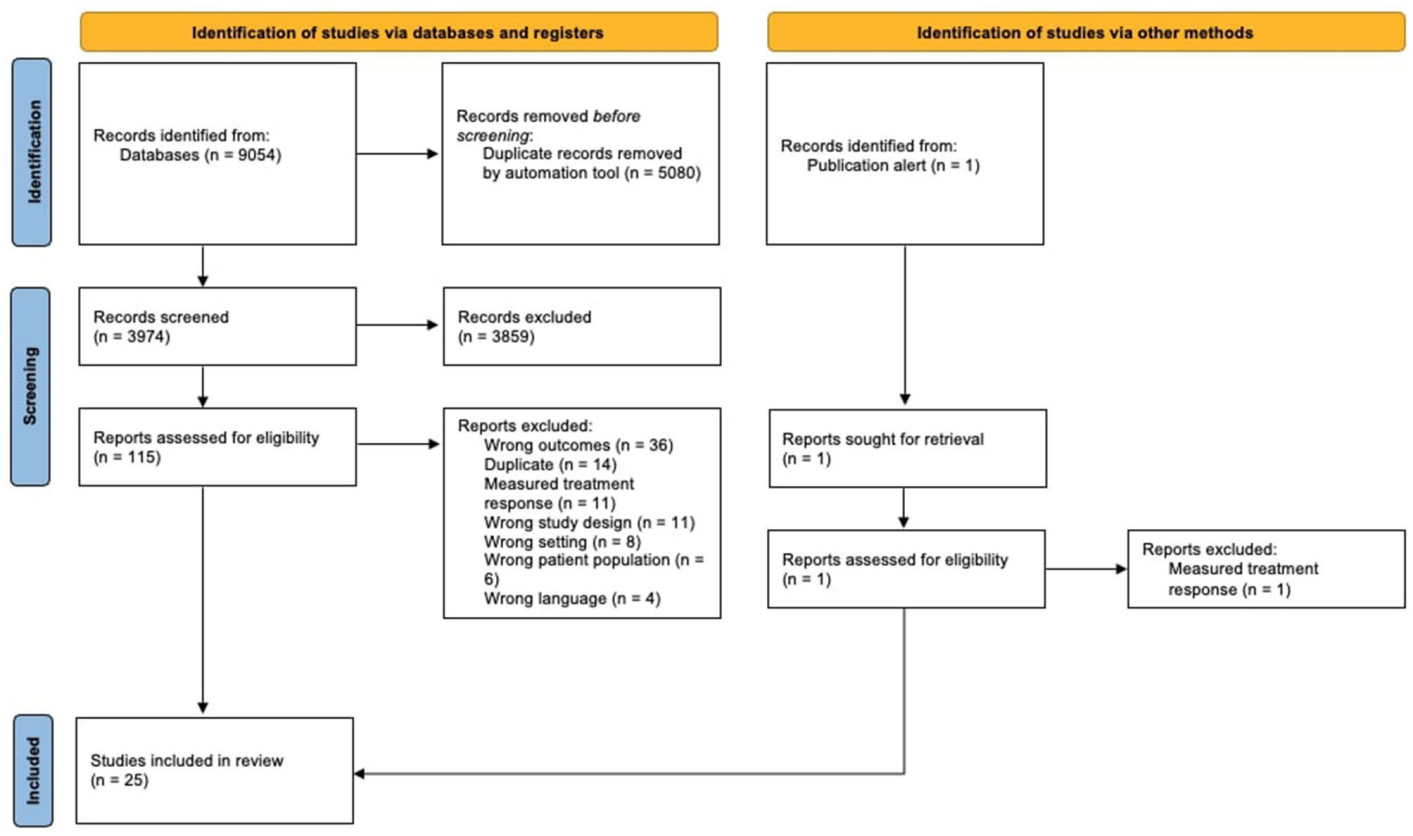
PRISMA (Preferred Reporting Items for Systematic Review and Meta-Analysis) flow diagram detailing study selection process.

Initially, 9055 search results were uploaded onto Covidence for screening. A combined 3974 relevant abstracts were identified after duplicates were automatically removed. Authors were contacted for full-texts or additional information. 115 full texts were assessed for eligibility prior to data extraction. Of these 115, one was added through an updated search alert. The final 25 studies underwent full data extraction and risk of bias assessment. Three studies were deemed to be predictive and 22 studies were diagnostic. The study selection process is illustrated in Figure 1 through a PRISMA diagram.

### Study Designs

Details of eligible studies are shown in Table 1. 13/25 (52%) studies were conducted in North America and the remainder were completed in Europe. 13/25 (52%) of the studies had <50 total participants. 14/25 (56%) studies followed the same patients during stable and PEx visits (paired samples). Two studies only used non-CF healthy controls instead of stable CF patients. The remaining studies collected data in both non-CF healthy individuals and stable CF patients. 8/25 (32%) of the eligible studies defined PEx diagnosis using previously published criteria (e.g., Fuchs, Rosenfeld, etc.) while the remaining studies used a variety of criteria such as physician-defined criteria, the decision to use intravenous antibiotics, or specific symptoms or tests. Most of the studies (22/25, 88%) in this present review examined metabolites for the diagnosis of PEx, as detailed in Table 1. Diagnostic validity concepts such as sensitivity and specificity of metabolites were only analyzed in three eligible studies.

### Biospecimen Types and Metabolite Methodologies

Biospecimen types and methods of metabolite identification and analysis varied among studies (Table 3). Out of the 25 included studies, 11/25 (44%) analyzed sputum, 10/25 (40%) plasma and/or serum, 5/25 (20%) exhaled breath condensate (EBC), and 2/25 (8%) urine samples. Within certain studies, multiple analytical platforms were used for various metabolites. For at least one included metabolite within a study, 4/25 (16%) used liquid chromatography with tandem mass spectrometry (LC-MS-MS), 3/25 (12%) used LC-MS, 4/25 (16%) used gas chromatography in some capacity with either MS or another apparatus, 6/25 (24%) used high-performance liquid chromatography (HPLC), and 4/25 (16%) used ultra-performance liquid chromatography (UPLC). Other analytical platforms were variable. A list of metabolites from the eligible studies categorized according to class as outlined by the Human Metabolome Database (HMDB) (35) is detailed in Table 2. Only named metabolites were included in the table, whereas compounds with only molecular formulas were not.

### Patient Characteristics From Included Studies

Patient characteristics of the included studies are detailed in Table 3. Seven studies focused on pediatric patients (<18 years old), nine studies included solely adult patients, and 8 studies included both populations studies included both populations. Few of the studies reported metabolites found in adults and pediatric patients separately. FEV_1_ values for both stable CF and PEx visits were not reported in every study. In Table 3, the FEV_1_ values represent only the PEx visits, which ranged from 32 to 82% across all studies.

### Risk of Bias Assessment

Using QUADAS-2, the risk of bias of eligible studies was determined by two independent reviewers (AVN and DH). Summary of the risk of bias and applicability concerns can be found in Supplementary Table 2 (Appendix). 10/25 (40%) of the studies had two or more domains within the risk of bias assessment rated as either “High risk” or “Unclear” and most studies demonstrated high risk of bias for the index test as the index test assessor, typically whoever was retrieving or analyzing the sample, was not blinded. Most included studies (18/25; 72%) did not pose any applicability concerns in any of the domains. Three studies (23–25) were deemed as high risk for applicability in the patient selection domain and only one (24) was deemed as high risk within the index test applicability domain. Since most of the studies were not explicitly designed to be diagnostic accuracy studies, some QUADAS-2 signaling questions were not applicable. In this case, the reviewers indicated the prompt as “Unclear.” Depending on the answers of the other domain-specific signaling questions, the reviewers would determine if there was a risk of bias despite the “Unclear” response.

### Biomarkers for Predicting and Diagnosing PEx

We identified 19 unique metabolites that were measured by two or more studies (Table 4). Metabolites were deemed to have potential for either prediction or diagnosis of PEx if: (1) the changes had consistent directionality in at least two studies within the same sample type (regardless of statistical significance) and at least one of the two studies compared PEx to stable CF samples as opposed to healthy controls as the latter comparison is more likely to be confounded by other factors; or (2) there was statistical significance within at least one study when comparing PEx to stable CF samples.

### Biomarkers to Predict PEx

Just three studies evaluated metabolites to predict PEx in CF and two were performed by the same research group (30, 31). In both studies, EBC lactic acid levels were higher in pre-PEx compared to stable CF samples. Zang et al. (30) also found statistical significantly higher levels of EBC pyroglutamic acid in pre-PEx compared to paired stable samples. Zang et al. (31) evaluated other metabolites from EBC including arginine, lysine, proline, glutamic acid, and 4-HCHC, but these were not found to be significantly different in pre-PEx compared to paired stable CF samples.

### Biomarkers to Diagnose PEx

Several metabolites from plasma and airway samples (sputum, EBC) were deemed to have potential as biomarkers to diagnose PEx. Plasma methionine was found to be lower in PEx compared to stable CF samples in two studies (7, 13), with one comparison being statistically significant (7). Three studies (7, 13, 16) found lower plasma arginine levels in PEx compared to healthy control or stable CF samples, but only the comparison to healthy controls was statistically significant (13). Plasma histidine, phenylalanine, and tryptophan levels were lower in PEx compared to healthy control samples in two separate studies (7, 13) but these were not compared to stable CF samples. Plasma carnitine (7, 31) and glutamate (7, 13) were significantly higher and plasma lysine and pyroglutamic acid levels were significantly lower in PEx compared to healthy control samples in one study (7) each but once again these were not compared to stable CF samples.

In terms of EBC, 4-HCHC and lactic acid levels were significantly higher in PEx compared to paired stable CF samples and both were evaluated in the same study (31). EBC NO levels were significantly lower in PEx compared to healthy control samples in one study (19) but was not compared to stable CF samples. For sputum, putrescine was consistently higher in PEx compared to healthly control and stable CF samples (15, 26) but only the comparison to healthy control samples was statistically significant. Sputum nitrate was consistently lower in PEx compared to stable CF samples in two studies, but the differences were not statistically significant (12, 16).

## Discussion

By applying metabolomic biomarkers to track disease activity, there is the potential to diagnose PEx earlier in its course. Thus, PEx management can be enacted earlier to prevent long-term and irreversible lung damage. While individual studies have evaluated metabolites to either predict or diagnose PEx in CF, this systematic review fills a gap in the literature by synthesizing all of the published studies to date to identify promising metabolites in need for further study.

### Heterogeneity and Bias in Study Designs

Despite some metabolites being evaluated in two or more studies, heterogeneity in study designs posed a challenge in synthesizing the results. While some studies used paired samples from individual patients during different disease states (i.e., stable vs. PEx), other studies used independent groups of healthy or CF patients to serve as stable controls. Use of independent groups is more vulnerable to confounding by factors other than disease state that might be driving the group differences in metabolite profiles.

Other considerations must also be made when studying the volatile nature of metabolites. One concern is the lack of a fasting protocol in most studies. The associated vitamins, minerals and macronutrients contained in food can impact the accuracy of test samples, especially when these same metabolites were the primary analyte of interest (36). The inconsistency across studies may impact the concentration of metabolites. Another consideration is the timeframe between sample collection and treatment. Antibiotics are routinely used for PEx treatment and may impact the metabolome during the initial treatment period. Thus, studies should ensure that samples are taken prior to any intervention and with uniform fasting protocols.

Another aspect to be considered is the reporting of blinding during index and reference testing. While it may be difficult to blind research coordinators collecting samples from CF or non-CF patients, those who process and analyze the samples should be blinded to the patient's disease state. One randomized pilot study did blind researchers during sample anaylsis (7).

Furthermore, existing studies have been conducted using a wide variety of sample sources and analytical methods to identify and quantify the metabolites. While there are advantages in collecting samples such as urine and blood due to ease of collection across most age groups and translation into clinical practice (37), sensitivity and specificity are potential concerns when monitoring lung disease status as detected metabolites could arise from other organs. Due to the wide array of metabolites and dynamic range of concentrations, no single analytical tool is capable of measuring all metabolites which is reflected in the diversity of platforms examined. As such, several of the included studies used two or more complementary analytical platforms. The most widely used analytical approaches were LC- and GC-MS, as well as ultra-performance LC (UPLC) and high-performance LC (HPLC), the latter separation techniques sometimes used in combination with MS to improve sensitivity and analyte resolution (38, 39). An advantage of LC-MS is that it does not require chemical pre-processing and can detect a broad range of metabolites which makes it ideal for untargeted discovery analysis. While GC-MS is more quantitative with excellent separation reproducibility and lower running costs than LC-MS, it is limited to the measurement of thermally stable and volatile compounds (40). Sample preparation and metabolite extraction differences across these platforms can pose a challenge in making comparisons across studies (41). As such, future studies must continue to explore the differences and similarities in metabolites across the various biological compartments and factor in the analytical platform used.

### Synthesis of Metabolite Results

Metabolites from the nitric oxide pathway have been the most extensively studied to date. Based on available data, it remains unclear if exhaled NO is lower during PEx compared to stable state as the studies performed to date have been relatively small and the findings have been inconsistent (17, 19). Metabolites of NO, including nitrate and nitrite, do not appear to be consistently higher or lower during PEx when sampled from saliva, plasma or urine but nitrates were lower in sputum in two studies but the differences were not statistically significant when compared to stable CF subjects (12, 16). Arginine, an amino acid that acts as a substrate to form NO, was not significantly higher in sputum during PEx (16) but another study demonstrated its potential to predict PEx when elevated in EBC (31). Contrary to the increase in airway sampling, plasma arginine was lower in CF PEx compared to healthy controls (13) but was not significantly lower than stable CF samples (16).

Carboxylic acids and derivatives have also been investigated in the context of PEx. EBC pyroglutamic acid levels were higher in pre-PEx compared to stable CF samples demonstrating the potential to predict PEx (30, 31). Plasma methionine levels were lower in PEx compared to stable CF samples and has the potential to diagnose PEx (7, 13). Plasma histidine, lysine, phenylalanine, pyroglutamic acid, and tryptophan levels were found to be lower and glutamic acid levels higher in CF PEx compared to healthy control samples but levels were not compared to stable CF (7, 13). This may reflect intrinsic differences between CF and healthy controls as opposed to differences related to PEx specifically as malnutrition in CF can lead to changes in the plasma amino acid profile compared to healthy subjects (42).

Within the organonitrogen compound class, sputum putrescine shows promise as being a diagnostic biomarker of PEx as levels were found to be higher in PEx compared to both healthy control and stable CF samples in two separate studies although the comparisons were not statistically significant due to the small sample sizes of the studies (15, 26). The potential role of ornithine-derived putrescine in PEx is unclear but it may play a role in smooth muscle regulation and anti-inflammatory processes (43). Within the organooxygen class, 4-hydroxycyclohexylcarboxylic acid (4-HCHC) from EBC was higher in PEx compared to stable CF samples. In terms of its potential role in CF pathophysiology, 4-HCHC is a rare organic acid involved in gut microbial and mammalian metabolism and its appearance in EBC is suggestive of gut-lung crosstalk with increased inflammation in both compartments (44).

Lactic acid, or lactate, has demonstrated both predictive and diagnostic potential in our review. When analyzed predictively, two studies from the same research group have found EBC lactic acid to be higher during pre-PEx compared to stable CF samples. Diagnostically, EBC and sputum lactic acid levels were higher in PEx compared to stable CF samples (26, 31). Lactic acid is a fermentation metabolite that might originate from anaerobic bacteria within the hypoxic airway environment. Anaerobic bacteria have been implicated in the pathophysiology of PEx (45).

### Limitations

Within this current review, there are several limitations that must be considered. Firstly, studies with statistically significant findings are more likely to be published which poses concern for publication bias similar to most systematic reviews. Furthermore, most conference abstracts were excluded during full-text review due to a lack of patient details, data presentation, and inability to assess risk of bias. Secondly, due to the heterogeneity of the study designs and objectives, we were unable to synthesize the results in a formal meta-analysis. Thus, we were limited to providing a tabulated summary of the studied metabolites and their respective directionality of change and the statistical significance of the change.

### Future Directions

With the evolving landscape of metabolomic research in CF, future studies must consider better standardization in study design, timing and method of metabolite collection and analysis, and reporting. Future studies that aim to test the diagnostic or predictive value of metabolite biomarkers in PEx should also ensure that data is collected during clearly defined disease states. The distinction between stable CF, pre-PEx, PEx, and pre-treatment and post-treatment states can be challenging but should be clearly outlined in future study designs. As well, consistency in fasting protocols across all sample types may be warranted.

Moreover, based on this review's search results, there were only two studies from the same research group that examined metabolites to help predict PEx (30, 31). This result highlights the need for more studies to consider prediction alongside diagnosis when characterizing biomarkers. While predicting PEx is understandably a more challenging clinical setting to apply biomarkers, this is the setting that will provide the most clinical utility as demonstrating differences in metabolites in settings in which there is a clear difference in clinical presentation (stable vs. PEx) is less useful but a reasonable starting point. Furthermore, comparison of metabolite levels to healthy control samples is less useful as differences may reflect differences between CF disease vs. healthy as opposed to disease activity related to PEx whereby a comparison to stable CF samples is much more relevant. Furthermore, metabolomic biomarkers for disease monitoring must also be re-evaluated in the context of CFTR modulator use as promising biomarkers, such as FeNO, can be influenced by CFTR modulators (46).

Lastly, it is important to consider how these studies can be translated into clinical practice in the long-term. For any promising metabolites to be used, they must undergo rigorous testing and validation as mandated by the Institute of Medicine to determine its appropriateness in clinical practice (47). Within this systematic review, we were unable to identify any metabolomic biomarkers that have undergone this process.

## Conclusion

Overall, this systematic review creates a foundation for future metabolomic biomarker research in PEx. While there are promising metabolites for predicting and diagnosing PEx that have been identified in multiple studies, further validation and exploration is needed. These studies should aim to standardize study design, metabolite collection, analysis, and reporting before clinical validation can be considered.

## Data Availability Statement

The original contributions presented in the study are included in the article/Supplementary Material, further inquiries can be directed to the corresponding author.

## Author Contributions

AVN and BQ conceptualized the research question, designed the study protocol, contributed to study screening, drafted the manuscript, revised for scientific content, and provided critical revisions. AVN, MB, and DH contributed to study screening and data extraction. AVN and DH completed risk of bias assessment. AVN, BQ, MB, and DH contributed to table design and formatting. BQ supervised the study and provided support. All authors reviewed and approved the final draft.

## Funding

BQ was supported by a Michael Smith Foundation for Health Research Scholar Award (#16414).

## Conflict of Interest

The authors declare that the research was conducted in the absence of any commercial or financial relationships that could be construed as a potential conflict of interest.

## Publisher's Note

All claims expressed in this article are solely those of the authors and do not necessarily represent those of their affiliated organizations, or those of the publisher, the editors and the reviewers. Any product that may be evaluated in this article, or claim that may be made by its manufacturer, is not guaranteed or endorsed by the publisher.
