# Supplementary material for: Metabolomic Biomarkers to Predict and Diagnose Cystic Fibrosis Pulmonary Exacerbations: A Systematic Review
Source: Front Pediatr. 2022 May 31;10:896439. doi: 10.3389/fped.2022.896439 (PMC9192952; doi:10.3389/fped.2022.896439)
Supplement: Supplementary file 1 [file Table_1.DOCX]

Supplementary Material

**Supplementary Table 1.** Example search strategy for MEDLINE via Ovid.

Database(s): **Ovid MEDLINE(R) ALL** 1946 to August 20, 2021

Search Strategy:

| **#** | **Searches** | **Results** |
| --- | --- | --- |
| 1 | Cystic Fibrosis/ | 36854 |
| 2 | Biomarkers/ | 306070 |
| 3 | pulmonary exacerbation.mp. or exp Respiratory Tract Infections/ or Pseudomonas Infections/ | 485223 |
| 4 | Pulmonary Disease, Chronic Obstructive/ or Disease Progression/ or exacerbation.mp. | 248614 |
| 5 | exp Metabolomics/ | 20708 |
| 6 | Magnetic Resonance Spectroscopy/ | 152842 |
| 7 | Volatile Organic Compounds/ | 11071 |
| 8 | 1 and 2 | 717 |
| 9 | 1 and 2 and 3 | 186 |
| 10 | 1 and 2 and 4 | 150 |
| 11 | 1 and 3 | 6375 |
| 12 | 1 and 3 and 5 | 8 |
| 13 | 1 and 5 | 48 |
| 14 | 1 and 7 | 21 |
| 15 | 1 and 2 and 6 | 6 |
| 16 | 3 and 5 | 164 |
| 17 | 1 and 6 | 56 |
| 18 | 3 or 4 | 724460 |
| 19 | 1 and 18 | 7397 |
| 20 | 2 or 5 | 322745 |
| 21 | 1 and 20 | 742 |
| 22 | 1 and 18 and 20 | 269 |

**Supplementary Table 2.** Risk of bias assessment using the QUADAS-2 tool.

| **Study** | **RISK OF BIAS** | | | | **APPLICABILITY CONCERNS** | | |
| --- | --- | --- | --- | --- | --- | --- | --- |
|  | **PATIENT SELECTION** | **INDEX TEST** | **REFERENCE STANDARD** | **FLOW AND TIMING** | **PATIENT SELECTION** | **INDEX TEST** | **REFERENCE STANDARD** |
| Alvarez 2017 | ☺ | ☹ | ☺ | ☺ | ☺ | ☺ | ☺ |
| Barr 2015 | ☺ | **?** | ☺ | ☺ | ☺ | **?** | ☺ |
| Cantin 2012 | **?** | ☹ | ☺ | ☺ | **?** | ☺ | ☺ |
| Felton 2021 | ☺ | ☹ | ☺ | ☺ | ☺ | ☺ | ☺ |
| Ghorbani 2015 | ☺ | ☹ | ☺ | ☺ | ☺ | ☺ | ☺ |
| Grasemann 1998 | ☹ | ☺ | ☺ | ☺ | **?** | ☺ | ☺ |
| Grasemann 2006 | ☺ | ☹ | ☺ | ☺ | ☺ | ☺ | ☺ |
| Grasemann 2011 | ☺ | ☹ | ☺ | ☺ | ☺ | ☺ | ☺ |
| Grasemann 2012 | ☺ | ☹ | ☺ | ☺ | ☺ | ☺ | ☺ |
| Hanusch 2020 | ☺ | ☹ | ☺ | **?** | ☺ | ☺ | ☺ |
| Ho 1998 | ☺ | ☹ | ☺ | ☺ | ☺ | ☺ | ☺ |
| Lagrange-Puget 2004 | ☺ | ☹ | ☺ | ☺ | ☺ | ☺ | ☺ |
| Linnane 1998 | ☺ | ☹ | ☺ | ☺ | ☺ | ☺ | ☺ |
| Lucca 2015 | ☺ | ☹ | ☺ | ☺ | ☺ | ☺ | ☺ |
| McGrath 1999 | ☺ | ☹ | ☺ | ☺ | ☺ | ☺ | ☺ |
| Montuschi 2011 | ☺ | ☺ | ☺ | ☺ | ☺ | ☺ | ☺ |
| Quinn 2016 | ☹ | ☹ | ☺ | ☹ | ☹ | ☺ | ☺ |
| Raghuvanshi 2020 | ☹ | ☹ | ☺ | ☹ | ☹ | ☹ | ☺ |
| Topcu 2020 | ☹ | ☹ | ☺ | ☹ | ☹ | ☺ | ☺ |
| Twomey 2013 | ☺ | ☹ | ☺ | ☹ | ☺ | ☺ | ☺ |
| Van Horck 2021 | ☺ | ☹ | ☺ | ☹ | ☺ | ☺ | ☺ |
| Vazquez 2016 | **?** | **?** | **?** | **?** | **?** | **?** | **?** |
| Wojewodka 2014 | ☺ | ☹ | ☺ | ☹ | ☺ | ☺ | ☺ |
| Zang 2017 | ☺ | ☹ | ☺ | ☺ | ☺ | ☺ | ☺ |
| Zang 2019 | ☺ | ☹ | ☺ | ☺ | ☺ | ☺ | ☺ |
| ☺ Low Risk          ☹ High Risk        **?** Unclear Risk | | | | | | | |
